# Hydrogen Sulfide Inhibits Inflammatory Pain and Enhances the Analgesic Properties of Delta Opioid Receptors

**DOI:** 10.3390/antiox10121977

**Published:** 2021-12-11

**Authors:** Aina Porta, Laura Rodríguez, Xue Bai, Gerard Batallé, Gerad Roch, Enric Pouso-Vázquez, Gianfranco Balboni, Olga Pol

**Affiliations:** 1 Grup de Neurofarmacologia Molecular, Institut d’Investigació Biomèdica Sant Pau, Hospital de la Santa Creu i Sant Pau, 08041 Barcelona, Spain; aina.porta@e-campus.uab.cat (A.P.); laura.rodriguezpe@e-campus.uab.cat (L.R.); xue.bai@e-campus.uab.cat (X.B.); gerard.batalle@e-campus.uab.cat (G.B.); gerard.roch@e-campus.uab.cat (G.R.); enrique.pousovazquez@e-campus.uab.cat (E.P.-V.); 2 Grup de Neurofarmacologia Molecular, Institut de Neurociències, Universitat Autònoma de Barcelona, 08193 Barcelona, Spain; 3 Unit of Pharmaceutical, Pharmacological and Nutraceutical Sciences, Department of Life and Environmental Sciences, University of Cagliari, 09042 Cagliari, Italy; gbalboni@unica.it

**Keywords:** analgesia, antioxidants, delta opioid receptors, inflammation, oxidative stress, pain

## Abstract

Chronic inflammatory pain is present in many pathologies and diminishes the patient’s quality of life. Moreover, most current treatments have a low efficacy and significant side effects. Recent studies demonstrate the analgesic properties of slow-releasing hydrogen sulfide (H_2_S) donors in animals with osteoarthritis or neuropathic pain, but their effects in inflammatory pain and related pathways are not completely understood. Several treatments potentiate the analgesic actions of δ-opioid receptor (DOR) agonists, but the role of H_2_S in modulating their effects and expression during inflammatory pain remains untested. In C57BL/6J male mice with inflammatory pain provoked by subplantar injection of complete Freund’s adjuvant, we evaluated: (1) the antiallodynic and antihyperalgesic effects of different doses of two slow-releasing H_2_S donors, i.e., diallyl disulfide (DADS) and phenyl isothiocyanate (P-ITC) and their mechanism of action; (2) the pain-relieving effects of DOR agonists co-administered with H_2_S donors; (3) the effects of DADS and P-ITC on the oxidative stress and molecular changes caused by peripheral inflammation. Results demonstrate that both H_2_S donors inhibited allodynia and hyperalgesia in a dose-dependent manner, potentiated the analgesic effects and expression of DOR, activated the antioxidant system, and reduced the nociceptive and apoptotic pathways. The data further demonstrate the possible participation of potassium channels and the Nrf2 transcription factor signaling pathway in the pain-relieving activities of DADS and P-ITC. This study suggests that the systemic administration of DADS and P-ITC and local application of DOR agonists in combination with slow-releasing H_2_S donors are two new strategies for the treatment of inflammatory pain.

## 1. Introduction

Chronic pain ranks among the 10 most prevalent diseases worldwide. It affects 18% of the general population in developing countries and is thus a serious public health problem [1]. Chronic inflammatory pain is characterized by multiple molecular, chemical, and physiological changes in the central and peripheral nervous system that cause important plasticity changes that perpetuate pain. The management of chronic pain is an unmet medical need and a big challenge in pain research. That is to say, although different therapeutic options are currently available most have limited efficacy and significant side effects [2,3]. Consequently, research into novel treatments for pain management is of great global importance.

Hydrogen sulfide (H_2_S) is a gaseous neurotransmitter that plays a key role in different physiological processes in the central and peripheral nervous system. The list of biological functions and signaling pathways modulated by the endogenous and exogenous administration of H_2_S is extensive and includes several processes, such as energetic dysfunctions, apoptosis, inflammatory responses, and oxidative stress [4]. However, the role of this neurotransmitter in chronic pain modulation is controversial, i.e., it has protective functions when released slowly, but exerts pro-inflammatory effects when released quickly [5]. In this context, recent studies proved that the exogenous administration of slow-releasing H_2_S donors, such as phenyl isothiocyanate (P-ITC) or allyl isothiocyanate (A-ITC), attenuated osteoarthritis and neuropathic pain, thus turning these compounds into potential therapeutical targets for the treatment of inflammatory pain [6,7,8]. Other investigations also demonstrated the antinociceptive effects of other slow-releasing H_2_S compounds, such as diallyl disulfide (DADS) and morpholin-4-ium 4-methoxyphenyl (morpholino) phosphinodithioate dichloromethane complex (GYY4137), in models of osteoarthritis or nerve injury-induced neuropathic pain [9,10]. These and other studies further revealed that the possible mechanisms involved in H_2_S-mediated antinociceptive actions in nociceptive, osteoarthritis, and neuropathic pain might be produced by opening the voltage-gated potassium channels, such as the Kv7 [6,7,10] and the ATP-sensitive (KATP) potassium channels [11,12]. However, the participation of these potassium channels in the possible antinociceptive effects of slow-releasing H_2_S donors during inflammatory pain has not been elucidated.

Several studies have also demonstrated that some of the H_2_S effects are mediated by triggering the Nrf2 transcription factor signaling pathway [5], which is highly implicated in defense mechanisms, redox homeostatic maintenance, and pain modulation [13,14]. The induction of Nrf2 activates the endogenous antioxidant system by promoting the transcription of various antioxidant enzymes, including heme oxygenase 1 (HO-1), NAD(P)H: quinone oxidoreductase 1 (NQO1), superoxide dismutase 1 (SOD-1), and glutathione S-transferase mu 1 (GSTM1), among others [15], that protect the organism against oxidative stress [16]. The contribution of the Nrf2/HO-1 signaling pathway in the analgesic actions of H_2_S during neuropathic pain is evidenced by the fact that their effects are reversed as a result of specific Nrf2 and HO-1 inhibitors, such as ML-385 and tin protoporphyrin IX (SnPP) [5]. Moreover, in a neuropathic pain model, treatment with NAHS, an H_2_S donor, upregulated the expression of Nrf2 and HO-1 in microglial cells of the spinal cord [17]. In this study, we examined the possible implication of the Nrf2/HO-1-NQO1 pathway in the pain-relieving effects of DADS and P-ITC during inflammatory pain.

Oxidative stress plays an essential role in the progress and preservation of chronic pain. The imbalance between oxidative and antioxidative changes induced by inflammation leads to oxidative stress. In consequence, the inhibition of the production of pro-inflammatory cytokines and reactive oxygen species limits the development of inflammatory pain [18]. Different studies revealed the protective role of H_2_S in oxidative stress in vitro [19] and in vivo [9].

Moreover, one study demonstrated that apoptosis precedes oxidative stress with the upregulation of Bcl2-associated X (BAX), an apoptosis-related protein [20]. Furthermore, in mice with acute liver injury, treatment with DADS inhibited apoptosis via the regulation of the PI3K/Akt signaling pathway [21]. In this study, the effects of DADS and P-ITC in the apoptotic and nociceptive reactions generated by peripheral inflammation were evaluated by measuring the expression of BAX and p-Akt in the paw.

Opioid receptors are key targets in pain modulation. There are three different major opioid receptor types: μ (MOR), κ, and δ (DOR), which are endogenously activated by peptides, including endorphins and dynorphins [22]. Nevertheless, it is well known that certain opioids present low efficacy, particularly MOR agonists, in the management of neuropathic pain and are accompanied by significant side effects [23,24]. In contrast, treatment with DOR agonists has recently been proposed as a more secure alternative to MOR agonists as their activation is associated with fewer unwanted effects, especially when administered locally [25]. However, DOR agonists, such as [D-Pen2,D-Pen5]-enkephalin (DPDPE) and H-Dmt-Tic-NH-CH(CH2-COOH)-Bid (UFP-512), have been shown to be moderately effective in chronic pain [26,27].

Therefore, novel strategies, such as their co-administration with other types of drugs, have been shown to enhance the antinociceptive effects of DOR agonists. In this way, the administration of Nrf2 or HO-1 activators potentiated the pain-relieving actions of DOR agonists in animals with chronic inflammatory pain [27,28]. Nevertheless, the effects of H_2_S inducers in terms of their palliative effects and the expression of DOR in animals with chronic inflammatory pain have not yet been evaluated.

Therefore, in a model of inflammatory pain induced by the subplantar administration of complete Freund’s adjuvant (CFA), our objectives were to evaluate: (1) the analgesic effects induced by the intraperitoneal administration of DADS and P-ITC; (2) their mechanism of action and effects in the oxidative, nociceptive, and apoptotic responses provoked by peripheral inflammation and (3) the effects of DADS and P-ITC in the antinociceptive actions and expression of DOR in paw tissues.

## 2. Materials and Methods

### 2.1. Animals

The experiments were carried out with 6–8-week-old male C57BL/6 mice (25–30 g), acquired at Envigo Laboratories (Barcelona, Spain), accommodated under 12/12 h light/dark conditions in a room with a controlled temperature of 22 °C and humidity of 66% until use. The animals had free access to food and water and were used after 7 days of acclimatization to the housing conditions. All experiments were conducted between 9:00 a.m. and 5:00 p.m. and executed in accordance with the guidelines of the European Commission’s directive (2010/63/EC) and the Spanish Law (RD 53/2013) regulating animal research, and they were approved by the local Committee of Animal Use and Care of the Autonomous University of Barcelona (ethical code: 9863). All efforts were made to diminish the suffering and number of animals employed in this study.

### 2.2. Induction of Inflammatory Pain

Inflammatory pain was induced by the subplantar injection of 30 μL of CFA (Sigma-Aldrich, St. Louis, MO, USA) in the right hind paw during short anesthetic procedures with isoflurane (3% induction, 2% maintenance), as used by our group [29]. Contralateral paws served as controls. Control animals were injected with the same volume of saline solution (NaCl 0.9%; SS). The development of mechanical allodynia and thermal hyperalgesia was evaluated by using the following sequence: von Frey filaments and plantar test. All animals were tested in each paradigm before and at 10 days after CFA-injection.

### 2.3. Mechanical Allodynia

Mechanical allodynia was evaluated by measuring the hind paw withdrawal response after stimulation with the von Frey filaments of different bending forces (ranging between 0.4 g and 3.0 g). Mice were placed in transparent tubes (20 cm high × 9 cm diameter) on a raised wire grid bottom, across which von Frey filaments (North Coast Medical, Inc., San Jose, CA, USA) were applied using the up/down paradigm described in [30]. The test started with the low bending force filament (0.4 g) and the strength of the next filaments was increased or decreased depending on the animal’s response. The threshold of the response was calculated from the sequence of filament strength used during the up/down procedure and using an Excel program (Microsoft Iberia SRL, Barcelona, Spain), which included curve fitting of the data. The 3.0 g filament was used as a cut-off. A clear paw withdrawal, licking, or shaking the paw was considered as a positive response. Both ipsilateral and contralateral hind paws were assessed.

### 2.4. Thermal Hyperalgesia

Thermal hyperalgesia was evaluated by assessing the paw withdrawal latency in response to beaming heat in the plantar test apparatus (Ugo Basile, Varese, Italy) [31]. Animals were placed in Plexiglas cylinders (20 cm high × 9 cm diameter) situated on a glass surface. The heat source was positioned under the plantar surface of the hind paws and activated with a light beam intensity until paw withdrawal, or the cut-off time of 12 s was reached. Mean paw withdrawal latencies were determined from the mean of three separate measurements of both the ipsilateral and contralateral hind paws.

In both tests, animals were habituated to the environment for 1 h before the test, so that they were quiet at the time of testing. All these experiments were performed by experimenters blinded to the experimental conditions.

### 2.5. Preparation of Whole-Cell Extracts and Western Blot Analysis

Animals were euthanized by cervical dislocation at 10 days after CFA or SS injection. The subplantar tissue samples of the hind paws on the ipsilateral side were extracted, quickly frozen, and maintained at −80 °C until use. The protein levels of 4-HNE, HO-1, NQO1, SOD1, GSTM1, p-Akt, BAX and DOR were analyzed. Tissue samples were homogenized in ice-cold lysis buffer (50 mM Tris-Base, 150 nM NaCl, 1% NP-40, 2 mM EDTA, 1 mM phenylmethylsulfonyl fluoride, 0.5 Triton X-100, 0.1% sodium dodecyl sulfate, 1 mM Na_3_VO_4_, 25 mM NaF, 0.5% protease inhibitor cocktail, 1% phosphatase inhibitor cocktail). All reagents were acquired from Sigma-Aldrich (St. Louis, MO, USA), except NP-40, which was obtained from Calbiochem (Darmstadt, Germany). After solubilization of crude homogenate for 1 h at 4 °C, samples were sonicated for 10 s and centrifuged at 4 °C for 20 min at 700× *g*. The supernatants (60 µg of total protein) were mixed with 4 × Laemmli loading buffer and loaded onto 4% stacking/12% separating sodium dodecyl sulfate polyacrylamide gels. Subsequently, the proteins were electrophoretically transferred onto a polyvinylidene fluoride membrane for 120 min and blocked with phosphate-buffered saline plus 5% nonfat dry milk or Tris-buffered saline with Tween 20 plus 5% nonfat dry milk or 5% bovine serum albumin for 75 min. Thereafter, the protein samples were incubated overnight at 4 °C with specific rabbit primary antibodies anti 4-HNE (1:150; Abcam, Cambridge, UK); HO-1 (1:150; Abcam, Cambridge, UK); NQO1 (1:200; Abcam, Cambridge, UK); GSTM1 (1:150; Novus Biologic, Littleton, CO, USA); SOD-1 (1:150; Novus Biologic, Littleton, CO, USA); phospho-Akt (1:200; Cell Signaling Technology, Danvers, MA, USA); total Akt (1:250; Cell Signaling Technology, Danvers, MA, USA); BAX (1:250; Cell Signaling Technology, Danvers, MA, USA); and DOR (1: 300; Abcam, Cambridge, UK). Blots were incubated for 1 h at room temperature with a horseradish peroxidase-conjugated anti-rabbit secondary antibody (GE Healthcare, Little Chalfont, UK). Proteins were detected using the chemiluminescence reagents (ECL kit; GE Healthcare, Little Chalfont, Buckinghamshire, UK). Lastly, immunoblots were quantified using a densitometric analysis with Image-J program (National Institutes of Health, Bethesda, MD, USA). We used a rabbit anti-glyceraldehyde-3-phosphate dehydrogenase (GAPDH) antibody (1:5000; Merck, Billerica, MA, USA) as a loading control.

### 2.6. Experimental Procedures

In the first set of experiments, the antinociceptive effects produced by the acute administration of different doses of DADS (25–200 µmols/kg), P-ITC (18.6–29 µmols/kg), or vehicle at 10 days after CFA-injection were evaluated (*n* = 6 animals per dose). Mice were tested at 1 h after DADS or P-ITC injection in accordance with our previous studies [8,10].

In other groups of animals, we evaluated the reversion of the antinociceptive effects produced by a high dose of DADS (200 µmols/kg) or P-ITC (29 µmols/kg) with the selective Kv7 potassium channel blocker XE-991 (12 µmols/kg), the KATP channel blocker (glibenclamide, 10 mg/kg), and the Nrf2 (ML-385, 25 mg/kg), HO-1 (SnPP, 10 mg/kg), or NQO1 (dicoumarol, 10 mg/kg) inhibitors (*n* = 6 animals per group). In all experiments, vehicle plus vehicle-treated animals were used as controls. The doses of DADS and P-ITC were selected according to the dose–response curves produced in this study and the doses of XE-991, glibenclamide, ML-385, SnPP, and dicoumarol were selected in accordance with previous studies [11,32,33,34].

CFA-injected mice treated with 200 µmols/kg of DADS, 29 µmols/kg of P-ITC, or vehicle were euthanized by cervical dislocation, and the protein levels of 4-HNE, HO-1, NQO1, GSTM1, SOD-1, p-AKT, BAX and DOR in the ipsilateral paw from mice with peripheral inflammation were evaluated by Western blot assay. In these experiments, saline-vehicle-treated mice were used as controls (*n* = 3–4 samples per group).

In other experiments, we studied the analgesic effects produced by the acute subplantar administration of different doses (50–300 µg) of two DOR agonists, DPDPE and UFP-512, at 30 min after their local administration (*n* = 6 animals per group). These doses were selected in accordance with other studies [27,28].

To analyze the potential enhancement of the analgesic effects of DOR agonists induced by H_2_S, we measured the antinociceptive actions generated by the intraperitoneal administration of low doses of DADS (25 μmol/kg) or P-ITC (18.6 μmol/kg) in combination with the subplantar administration of a low dose (50 μg) of DPDPE or UFP-512. The doses of DPDPE and UFP-512 were extracted from the corresponding dose–response curves. DADS and P-ITC were administered 30 min before the subplantar injection of DPDPE or UFP-512, and tests were performed 30 min later (*n* = 6 animals per group).

### 2.7. Drugs

P-ITC, DADS, DPDPE, and glibenclamide, obtained from Sigma-Aldrich (St. Louis, MO, USA), and UFP-512, synthesized by Balboni et al., (2002) [35], were dissolved in SS. XE-991, purchased in Tocris Bioscience (Ellisville, MO, USA), ML-385 and dicoumarol, obtained from Eurodiagnostico S.L, (Madrid, Spain) and SnPP, purchased in Frontier scientific (Livchem GmbH & Co, Frankfurt, Germany), were dissolved in dimethyl sulfoxide (1% SS).

P-ITC and DADS were intraperitoneally administered at 10 mL/kg 1 h before the tests, while XE-991, glibenclamide, ML-385, SnPP, and dicoumarol were also intraperitoneally administered at 10 mL/kg 30 min before the tests, in accordance with previous studies [10,11,32,34]. DPDPE and UFP-512 were administered via subplantar injection in a final volume of 30 μL 30 min before the behavioral tests, in accordance with our previous studies [27,28].

All drugs were freshly prepared just before being administered. For each group treated with a drug, the respective control group received the same volume of vehicle.

### 2.8. Statistical Analyses

Data are expressed as mean values ± standard error of the mean (SEM). We used the Graph Pad Prism program (version 8 for Windows) for the statistical analysis.

The analysis of the effects in each behavioral test produced by different doses of DADS, P-ITC, DPDPE, UFP-512, or vehicle was conducted using a one-way ANOVA and a Student–Newman–Keuls test. The ED_50_ of the drugs was calculated by linear regression analysis using GraphPad Prism (version 8 for Windows). The antinociceptive actions of DADS or P-ITC administered alone and in combination with DPDPE or UFP-512 were also analyzed using a one-way ANOVA followed by a Student–Newman–Keuls test.

The effects produced by the co-administration of H_2_S donors with specific potassium channel blockers or Nrf2/HO-1/NQO1 antagonists were analyzed using a one-way ANOVA followed by a Student–Newman–Keuls test.

In these experiments, antinociception in von Frey filaments and plantar tests is expressed as the percentage of the maximal possible effect, in which the test latencies pre (baseline) and post drug administration were compared and calculated according to the following equation:

Maximal possible effect (%) = [(drug − baseline)/(cut-off − baseline)] × 100

Changes in the protein levels of 4-HNE, HO-1, NQO1, SOD1, GSTM1, p-AKT, BAX and DOR in the paw tissues were also examined using a one-way ANOVA and the corresponding Student–Newman–Keuls test. A *p* < 0.05 was considered statistically significant.

## 3. Results

### 3.1. Effects of the Acute Administration of DADS and P-ITC in the Mechanical Allodynia and Thermal Hyperalgesia Induced by CFA

**Figure 1:**
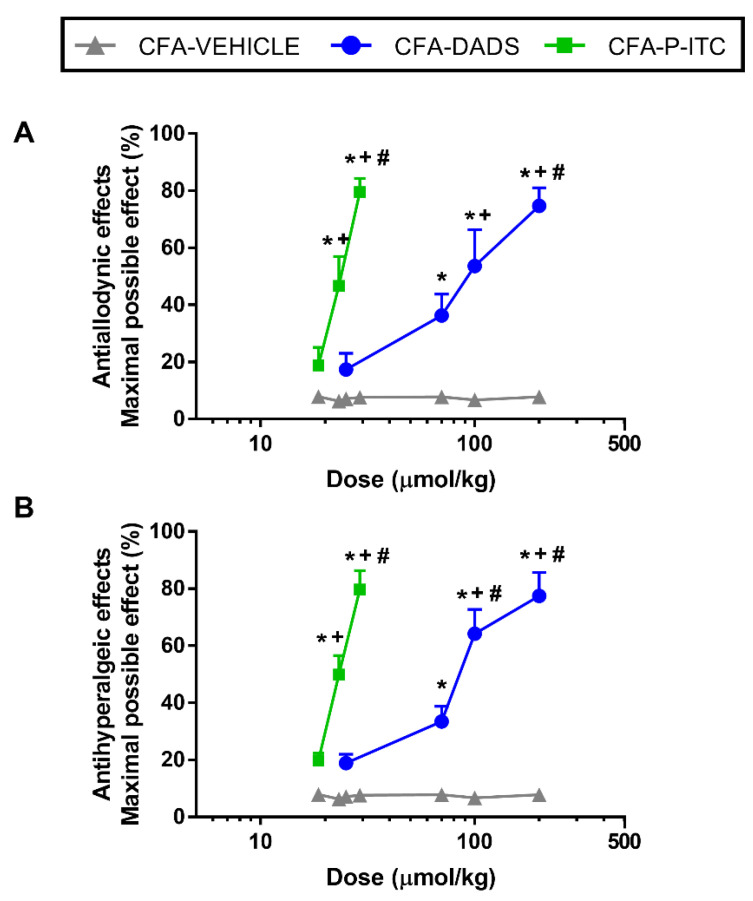
Effects of the acute intraperitoneal administration of DADS and P-ITC on the mechanical allodynia and thermal hyperalgesia induced by peripheral inflammation. Effects of different doses (logarithmic axis) of DADS and P-ITC (µmols/kg) on mechanical allodynia (**A**) and thermal hyperalgesia (**B**) induced by CFA in the ipsilateral paw of mice. For each test, * denotes significant differences vs. mice administered with vehicle, + denotes significant differences vs. mice administered with 25 µmols/kg of DADS or 18.6 µmols/kg of P-ITC, respectively, and # indicates significant differences vs. mice treated with 70 µmols/kg of DADS or 23.2 µmols/kg of P-ITC, respectively (*p* < 0.05; one-way ANOVA followed by Student–Newman–Keuls test). The results are shown as mean values of the maximal possible effect (%) ± SEM; *n* = 6 animals per dose and treatment.

We evaluated the effects of the acute intraperitoneal administration of 25, 70, 100, and 200 µmols/kg of DADS or 18.6, 23.2, and 29 µmols/kg of P-ITC or their corresponding vehicles on CFA-induced mechanical allodynia and thermal hyperalgesia at 10 days after inducing inflammation. Our results showed that the administration of DADS inhibited CFA-induced mechanical allodynia (Figure 1A) and thermal hyperalgesia (Figure 1B) in a dose-dependent manner and reached the maximum effect at the dose of 200 µmols/kg. Similar results were observed with P-ITC, which also inhibited mechanical allodynia (Figure 1A) and thermal hyperalgesia (Figure 1B) in a dose-dependent manner, having a maximal effect at a dose of 29 µmols/kg.

The mechanical antiallodynic and thermal antihyperalgesic effects produced by different doses of DADS, i.e., 70, 100, and 200 µmols/kg, in mice with inflammatory pain were greater than those produced by the vehicle (*p* < 0.0001; one-way ANOVA, followed by the Student–Newman–Keuls test). In both tests, the effects of DADS at 100 and 200 µmol/kg were greater than those produced by 25 and/or 70 µmols/kg.

Similarly, the antiallodynic and antihyperalgesic effects generated by 23.2 and 29 µmols/kg of P-ITC in mice with CFA-induced peripheral inflammation were greater than those produced by vehicle (*p* < 0.0001; one-way ANOVA, followed by the Student–Newman–Keuls test). In addition, in both tests, the effects of 29 μmol/kg P-ITC were greater than those produced by 18.6 and 23.2 μmol/kg (*p* < 0.001; one-way ANOVA, followed by the Student–Newman–Keuls test) and the effects of 23.2 μmol/kg P-ITC were also greater than those produced by 18.6 µmols/kg (*p* < 0.001; one-way ANOVA, followed by the Student–Newman–Keuls test).

Neither the vehicle nor DADS or P-ITC exhibited any antiallodynic or antihyperalgesic effect in the contralateral paws (data not shown).

Moreover, by analyzing the ED_50_ values of DADS and P-ITC, the data revealed that the potency of P-ITC in the inhibition of the mechanical and thermal sensitivity induced by peripheral inflammation was between 3.9 and 3.8 times greater than that of DADS (Table 1), indicating that the synthetic H_2_S donor (P-ITC) is more potent than the natural (DADS) donor in inhibiting inflammatory pain.

### 3.2. Reversion of the Antiallodynic and Antihyperalgesic Effects Induced by High Doses of DADS or P-ITC with Their Co-Administration with Two Potassium Channels Inhibitors

To evaluate the possible mechanisms implicated in the antinociceptive effects of DADS and P-ITC during inflammatory pain, the effects of the acute intraperitoneal administration of 200 µmols/kg of DADS or 29 µmols/kg of P-ITC in combination with a selective Kv7 potassium channel blocker (XE-991) or a KATP channel blocker (glibenclamide) on the mechanical allodynia and thermal hyperalgesia caused by peripheral inflammation were assessed.

**Figure 2:**
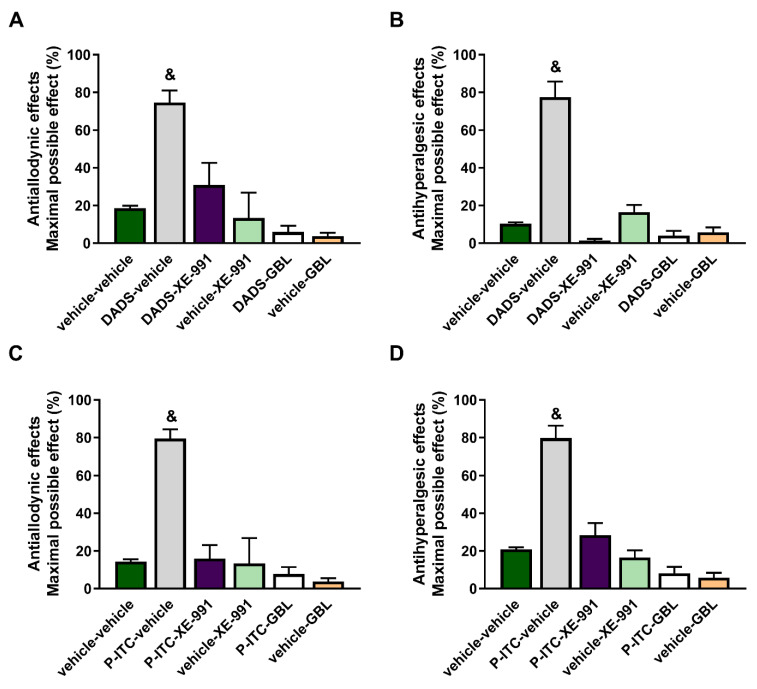
Effects of the administration of DADS or P-ITC alone or in combination with XE-991 or glibenclamide on the mechanical allodynia and thermal hyperalgesia induced by CFA. The antiallodynic (**A**,**C**) and antihyperalgesic (**B**,**D**) effects produced by DADS at 200 µmols/kg or P-ITC at 29 µmols/kg in the ipsilateral paws of mice with inflammatory pain co-treated with a Kv7 potassium channel blocker (XE-991, 12 µmols/kg), a KATP channel blocker (glibenclamide, 10 mg/kg), or vehicle are shown. For each test and treatment evaluated & indicates significant differences vs. the other groups (*p* < 0.05, one-way ANOVA followed by Student–Newman–Keuls test). Results are represented as mean values of the maximal possible effect (%) ± SEM values; *n* = 6 animals per experimental group.

Our results showed that both XE-991 and glibenclamide reversed the antiallodynic effects produced by 200 µmol/kg of DADS (*p* < 0.001, one-way ANOVA vs. vehicle + vehicle-treated mice) (Figure 2A) and 29 µmol/kg of P-ITC (*p* < 0.001, one-way ANOVA vs. vehicle + vehicle-treated mice) (Figure 2C), and they also reversed the thermal antihyperalgesic effects of DADS (*p* < 0.001, one-way ANOVA vs. vehicle + vehicle-treated mice) (Figure 2B) and P-ITC (*p* < 0.001, one-way ANOVA vs. vehicle + vehicle-treated mice) (Figure 2D) during inflammatory pain. The administration of XE-991 and glibenclamide alone did not produce any significant effect in the ipsilateral paws of CFA-injected animals. Moreover, XE-991 and glibenclamide administered alone or in combination with DADS or P-ITC did not produce any effect in the contralateral paws of CFA-injected mice (data not shown).

### 3.3. Reversion of the Antiallodynic and Antihyperalgesic Effects Induced by High Doses of DADS or P-ITC with Their Co-Administration with Specific Inhibitors of the Nrf2/HO-1-NQO1 Signaling Pathway

To study the probable participation of the Nrf2/HO-1/NQO1 signaling pathway in the analgesic actions of DADS and P-ITC, the effects of the co-administration of 200 µmols/kg of DADS or 29 µmols/kg of P-ITC with a selective Nrf2 (ML-385, 25 mg/kg), HO-1 (SnPP, 10 mg/kg), or NQO1 (dicoumarol, 10 mg/kg) inhibitor on the mechanical allodynia and thermal hyperalgesia caused by peripheral inflammation were evaluated.

**Figure 3:**
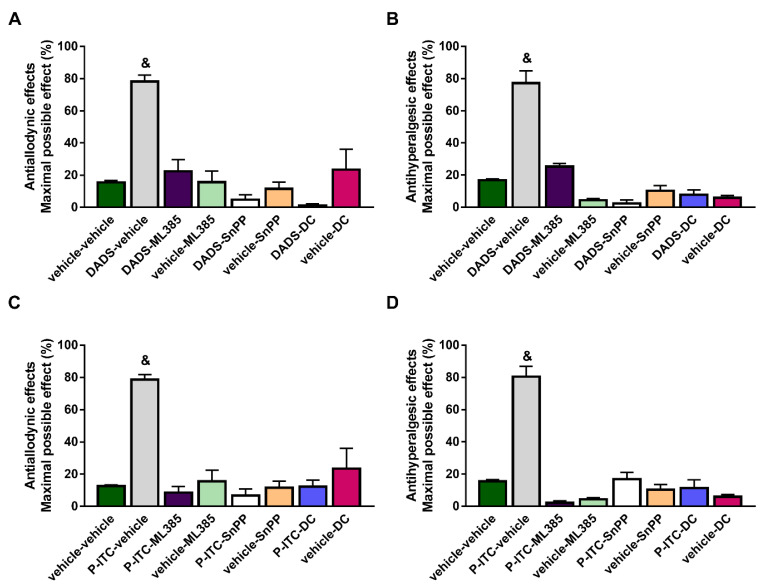
Effects of DADS and P-ITC administered alone or in combination with inhibitors of the Nrf2/HO-1-NQO1 signaling pathway on the mechanical allodynia and thermal hyperalgesia induced by CFA. The effects on the mechanical allodynia (**A**,**C**) and thermal hyperalgesia (**B**,**D**) in the ipsilateral paws of mice with inflammatory pain treated with DADS at 200 µmols/kg or P-ITC at 29 µmols/kg alone or co-administered with a Nrf2 inhibitor (ML-385, 25 mg/kg), an HO-1 inhibitor (SnPP, 10 mg/kg), or a NQO1 inhibitor (dicoumarol, DC, 10 mg/kg) are shown. For each test and treatment evaluated & indicates significant differences vs. the other groups (*p* < 0.05, one-way ANOVA followed by Student–Newman–Keuls test). Results are represented as mean values of the maximal possible effect (%) ± SEM values; *n* = 6 animals per experimental group.

Our results demonstrated the reversion of the antiallodynic (Figure 3A,C) and antihyperalgesic effects (Figure 3B,D) induced by DADS or P-ITC in the ipsilateral paw of mice with inflammatory pain after their co-administration with ML-385, SnPP, or dicoumarol (*p* < 0.0001, one-way ANOVA followed by Student–Newman–Keuls). In both tests, the administration of ML-385, SnPP, or dicoumarol alone did not produce any significant effect in the ipsilateral paws of CFA-injected animals.

In addition, treatment with ML-385, SnPP, or dicoumarol administered alone or in combination with DADS or P-ITC did not induce any effect in the contralateral paws of CFA-injected mice (data not shown).

### 3.4. Effects of Treatment with DADS and P-ITC on the Expression of 4-HNE in the Paws of Animals with Peripheral Inflammation

**Figure 4:**
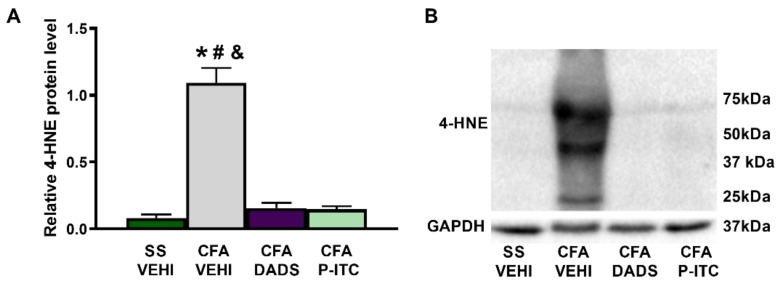
Effects of treatment with DADS and P-ITC on the expression of 4-HNE in the ipsilateral paws of animals with peripheral inflammation. The protein levels of 4-HNE in the ipsilateral paws of CFA-injected mice treated with vehicle (CFA-VEHI), DADS (CFA-DADS), or P-ITC (CFA-P-ITC) are shown (**A**). The expression of this protein in the ipsilateral paws of mice treated with saline plus vehicle (SS-VEHI) is also represented as controls (**A**). Representative blots for 4-HNE and GAPDH are shown (**B**). For each column, * indicates significant differences when compared with saline plus vehicle-treated animals, # indicates differences when compared with CFA-injected mice treated with DADS, and & indicates significant differences when compared with CFA-injected mice treated with P-ITC (*p* < 0.05, one-way ANOVA followed by Student–Newman–Keuls test). Data are expressed as mean ± SEM; *n* = 3–4 samples.

To measure the effects of DADS and P-ITC on the oxidative stress induced by CFA, we evaluated their actions in the expression of the oxidative marker 4-HNE. Our results showed an increase in the expression of 4-HNE in the paw of CFA-injected mice treated with vehicle (*p* < 0.0001, one-way ANOVA as compared with saline plus vehicle treated mice), which was completely reversed in DADS- or P-ITC-treated mice (Figure 4).

### 3.5. Effects of Treatment with DADS and P-ITC on the Expression of HO-1, NQO1, SOD-1, and GSTM1 in the Paws of Mice with Peripheral Inflammation

**Figure 5:**
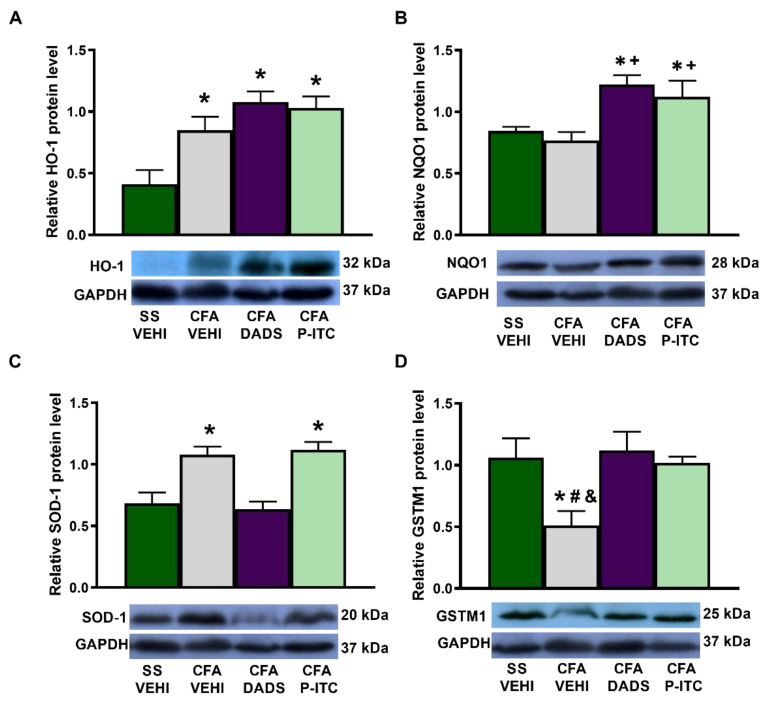
Effects of treatment with DADS and P-ITC on the expression of HO-1, NQO1, SOD-1, and GSTM1 in the paw tissues of animals with peripheral inflammation. The protein levels of HO-1 (**A**), NQO1 (**B**), SOD-1 (**C**), and GSTM1 (**D**) in the ipsilateral paw of CFA-injected mice treated with vehicle (CFA-VEHI), DADS (CFA-DADS) or P-ITC (CFA-P-ITC) are shown. The expression of these proteins in the paw tissues of mice treated with saline + vehicle (SS-VEHI) as controls is also shown. For each protein, * indicates significant differences when compared with SS plus vehicle-treated mice, + indicates significant differences when compared with CFA plus vehicle-treated animals, # indicates significant differences when compared with CFA plus DADS-treated mice, and & indicates significant differences when compared with CFA plus P-ITC-treated mice (*p* < 0.05, one-way ANOVA followed by Student–Newman–Keuls test). Representative examples of blots for HO-1, NQO1, SOD-1, GSTM1, and GAPDH are shown. Data are expressed as mean ± SEM; *n* = 3–4 samples.

To assess the implication of the antioxidant system in the antinociceptive effects of slow-releasing H_2_S donors during inflammatory pain, we evaluated the expression of HO-1, NQO1, SOD-1, and GSTM1 in the ipsilateral paw of CFA-injected mice treated with DADS or P-ITC. While peripheral inflammation enhanced the paw HO-1 (*p* < 0.0022, one-way ANOVA, as compared with saline + vehicle-treated animals) (Figure 5A) and SOD-1 expression (*p* < 0.0036, one-way ANOVA, as compared with saline + vehicle-treated animals) (Figure 5C), it decreased the paw expression of GSTM1 (*p* < 0.0313, one-way ANOVA, as compared with saline + vehicle-treated animals) (Figure 5D). Non-changes in the NQO1 levels were detected in the paws of CFA injected mice treated with vehicle (Figure 5B). Moreover, while treatment with DADS and P-ITC maintained the high levels of HO-1 (*p* < 0.0022, one-way ANOVA, as compared with saline + vehicle-treated animals) (Figure 5A), both treatments enhanced the expression of NQO1 (*p* < 0.0043, one-way ANOVA, as compared with saline + vehicle and CFA + vehicle treated animals) (Figure 5B). Regarding the SOD-1 levels, only P-ITC treatment maintained the high levels of this protein (*p* < 0.0036, one-way ANOVA, as compared with saline + vehicle-treated animals) (Figure 5C) and both treatments normalized the decreased expression of GSTM1 provoked by CFA (*p* < 0.0313, one-way ANOVA, as compared with CFA plus vehicle-treated animals) (Figure 5D).

### 3.6. Effects of Treatment with DADS and P-ITC on the Expression of p-AKT, BAX, and DOR in the Paws of Animals with Peripheral Inflammation

**Figure 6:**
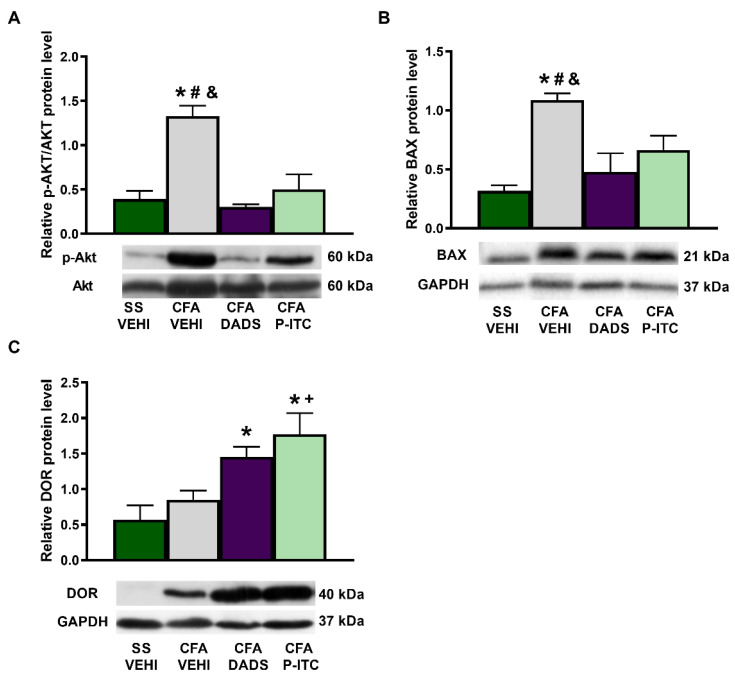
Effects of treatment with DADS and P-ITC on the expression of p-AKT, BAX, and DOR in the paw tissues of animals with peripheral inflammation. The protein levels of p-AKT/AKT (**A**), BAX (**B**), and DOR (**C**) in the ipsilateral paw of CFA-injected mice treated with vehicle (CFA-VEHI), DADS (CFA-DADS), or P-ITC (CFA-P-ITC) are shown. The expression of these proteins in the paw tissues of mice treated with saline + vehicle (SS-VEHI), as controls, is also shown. For each protein, * indicates significant differences when compared with saline plus vehicle-treated mice, + indicates significant differences when compared with CFA plus vehicle-treated animals, # indicates significant differences when compared with CFA plus DADS-treated mice, and & indicates significant differences when compared with CFA plus P-ITC-treated mice (*p* < 0.05, one-way ANOVA followed by Student–Newman–Keuls test). Representative examples of blots for p-AKT, AKT, BAX, DOR, and GAPDH are shown. Data are expressed as mean ± SEM; *n* = 3–4 samples.

To assess the role played by DADS and P-ITC in the nociceptive and apoptotic reactions induced by CFA, we evaluated the expression of p-AKT and BAX in the ipsilateral paw of CFA-injected mice treated with these H_2_S donors. CFA increased the paw expression of p-AKT/AKT (*p* < 0.0006, one-way ANOVA) (Figure 6A) and BAX (*p* < 0.0096, one-way ANOVA) (Figure 6B) as compared with their respective saline + vehicle-treated animals. Treatment with DADS and P-ITC normalized the upregulation of both p-AKT and BAX induced by peripheral inflammation.

To assess the probable mechanisms involved in the enhanced antinociceptive effects of DPDPE and UFP-512 observed in mice co-treated with DADS and P-ITC, the expression of DOR in the paws of CFA-injected mice was also evaluated. Our data revealed that, although the DOR protein levels were not altered by CFA injection, they were significantly increased in DADS- and P-ITC-treated animals (*p* < 0.0191, one-way ANOVA as compared with saline + vehicle- and/or CFA + vehicle-treated animals) (Figure 6C).

### 3.7. Effects of the Acute Administration of DPDPE and UFP-512 in the Mechanical Allodynia and Thermal Hyperalgesia Induced by CFA

**Figure 7:**
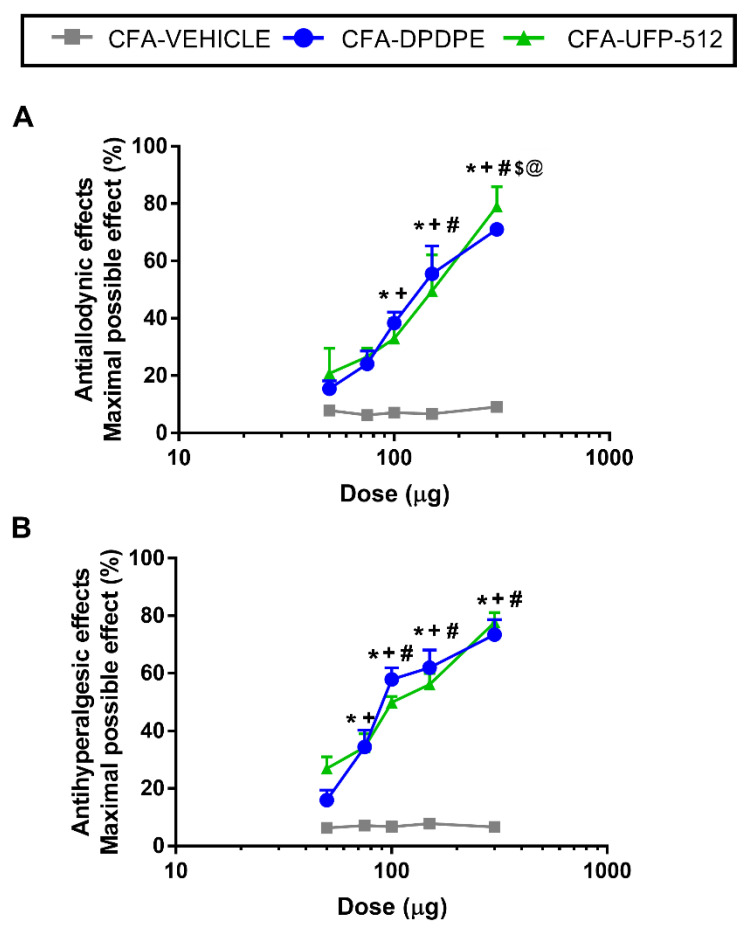
Effects of the subplantar administration of DPDPE or UFP-512 in the mechanical allodynia and thermal hyperalgesia induced by peripheral inflammation. Mechanical antiallodynic (**A**) and thermal antihyperalgesic (**B**) effects of different doses (logarithmic axis) of DPDPE or UFP-512 are shown. For each dose evaluated, * indicates significant differences vs. animals treated with vehicle, + indicates significant differences vs. the effect produced by 50 μg of DPDPE or UFP-512, # denotes significant differences vs. the effect produced by 70 μg of DPDPE or UFP-512, $ indicates significant differences vs. the effect produced by 100 μg of DPDPE or UFP-512, and @ indicates significant differences vs. the effect produced by 150 μg of DPDPE or UFP-512 (*p* < 0.05; one-way ANOVA, followed by Student–Newman–Keuls test). Data are expressed as mean values of maximal possible effect (%) ± SEM (*n* = 6 animals per dose).

We evaluated the effects of the acute subplantar administration of 50, 75, 100, 150, and 300 μg of DPDPE or UFP-512 in the mechanical allodynia and thermal hyperalgesia induced by CFA. The local administration of DPDPE or UFP-512 inhibited the mechanical allodynia (Figure 7A) and thermal hyperalgesia (Figure 7B) in a dose-dependent manner, in which the maximum effect was produced by 300 μg of DPDPE or UFP-512.

Specifically, the inhibition of the mechanical allodynia produced by high doses of DPDPE or UFP-512 (100, 150, and 300 μg) was significantly greater than the effects produced by low doses of these drugs (50 or 75 μg) or vehicle. Moreover, the three high doses produced increasing progressive effects with significant differences between them (*p* < 0.001; one-way ANOVA, followed by Student–Newman–Keuls test).

The inhibition of the thermal hyperalgesia produced by high doses (100, 150, and 300 μg) of DPDPE or UFP-512 exhibited no significant difference between them but were different than those produced by 50 and 70 µg of these drugs or vehicle (*p* < 0.001; one-way ANOVA, followed by Student–Newman–Keuls test). The antihyperalgesic effects of 70 μg DPDPE or UFP-512 were greater than those produced by 50 µg of these drugs or the vehicle (*p* < 0.001; one-way ANOVA, followed by Student–Newman–Keuls test). Neither the vehicle nor DPDPE or UFP-512 administration produced any antinociceptive effects in the contralateral paws (data not shown).

Moreover, the ED_50_ values of DPDPE and UFP-512 revealed that the potency of DPDPE in the inhibition of the mechanical and thermal sensitivity induced by peripheral inflammation was approximately 1.6 times greater than that produced by UFP-512 (Table 2).

### 3.8. Enhancement of the Antinociceptive Actions Generated by the Subplantar Administration of DPDPE and UFP-512 during Inflammatory Pain in Mice Treated with DADS or P-ITC

**Figure 8:**
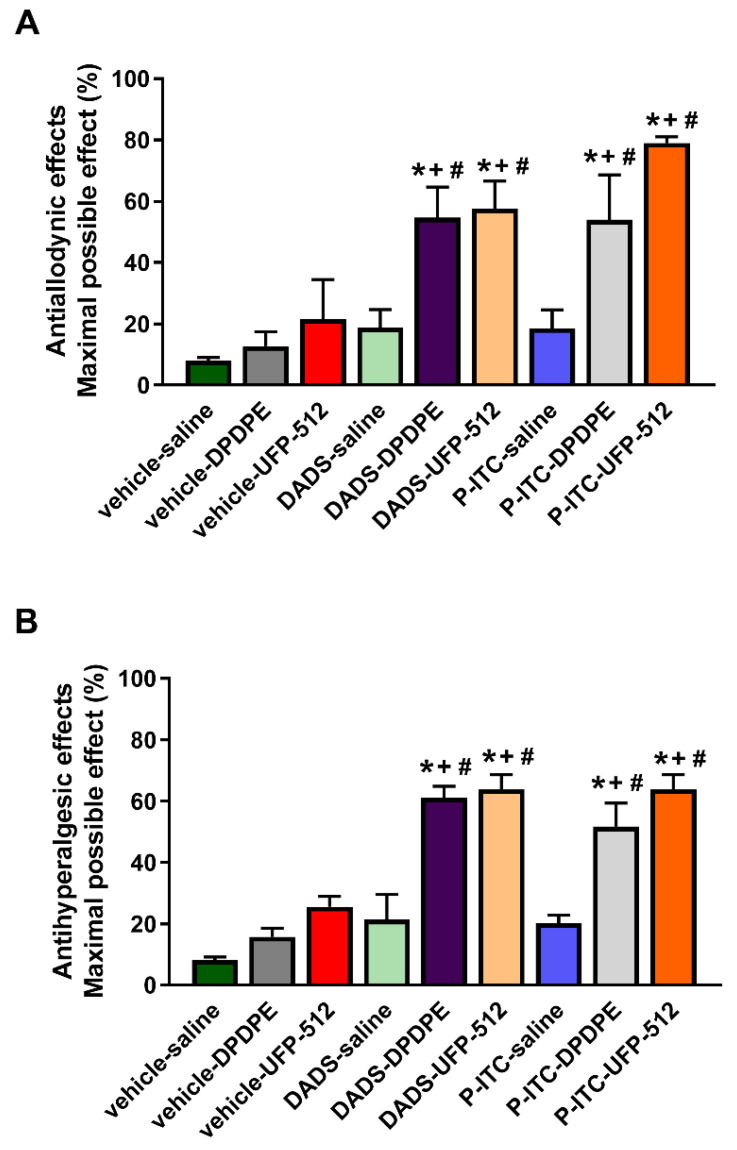
Effects of the acute co-administration of H_2_S donors with DOR agonists on the mechanical allodynia and thermal hyperalgesia caused by peripheral inflammation. Effects of the acute intraperitoneal administration of 25 μmol/kg of DADS or 18.6 μmol/kg of P-ITC alone and in combination with the subplantar administration of 50 μg of DPDPE, UFP-512, or saline in the mechanical allodynia (**A**) and thermal hyperalgesia (**B**) caused by CFA in the ipsilateral paw are shown. For each test, * indicates significant differences vs. vehicle plus saline-treated animals, + indicates significant differences vs. vehicle plus DPDPE- or UFP-512-treated mice, and # represents significant differences when compared with DADS or P-ITC plus saline treated animals (*p* < 0.05; one-way ANOVA, followed by the Student–Newman–Keuls test). Data are shown as mean values of the maximal possible effect (%) ± SEM (*n* = 6 animals per experimental group).

We evaluated the antinociceptive effects produced by the subplantar administration of a low dose (50 µg) of DPDPE or UFP-512 alone and in combination with the acute intraperitoneal administration of low doses of DADS (25 μmol/kg) or P-ITC (18.6 μmol/kg) in the mechanical allodynia (Figure 8A) and thermal hyperalgesia (Figure 8B) induced by peripheral inflammation.

The results demonstrated that the co-administration of DADS or P-ITC with DPDPE or UFP-512 significantly enhanced the mechanical antiallodynic actions generated by both DOR agonists (*p* < 0.001, one-way ANOVA followed by a Student–Newman–Keuls test; as compared with their respective control groups treated with vehicle plus saline, DPDPE, or UFP-512, and with mice treated with DADS or P-ITC plus saline).

Similar results were observed regarding the thermal antihyperalgesic effects. That is, DPDPE or UFP-512 co-administered with DADS or P-ITC exhibited significantly greater effects than their respective control groups treated with vehicle plus saline, DPDPE, or UFP-512, and with animals treated with DADS or P-ITC plus saline (*p* < 0.001, one-way ANOVA followed by a Student–Newman–Keuls test).

The administration of vehicle or any of the tested combinations did not produce any action in the contralateral paw in mechanical allodynia nor in thermal hyperalgesia (data not shown).

## 4. Discussion

The present study revealed that the administration of two slow-releasing H_2_S donors, DADS and P-ITC, inhibited the nociceptive responses caused by peripheral inflammation in a dose-dependent manner and potentiated the analgesic activities of DOR agonists. These effects are principally mediated by reducing oxidative stress and the nociceptive and apoptotic reactions generated by peripheral inflammation and enhancing the expression of DOR in paw tissues. This study also demonstrated the participation of potassium channels and the Nrf2/HO-1-NQO1 signaling pathway in the analgesic effects of P-ITC and DADS during peripheral inflammation.

The role of H_2_S in pain modulation is controversial. Several studies showed the contradictory nature of its functions, i.e., while fast H_2_S releasers appear to have pro-nociceptive functions, recent studies showed that substances capable of releasing H_2_S slowly, simulating H_2_S release in vivo, exert antinociceptive effects in different pain models, such as neuropathic or osteoarthritis pain [7,8,10,36]. In accordance, our results further revealed that the intraperitoneal administration of DADS and P-ITC inhibited the mechanical allodynia and thermal hyperalgesia induced by peripheral inflammation in a dose-dependent manner, whereas higher doses of DADS (200 µmols/kg) or P-ITC (29 µmols/kg) inhibited the CFA-induced nociception, with a maximal effect of 80%. Our data also revealed the greater effectiveness of P-ITC vs. DADS in terms of inhibiting CFA-induced allodynia and hyperalgesia, indicating that the synthetic compound (P-ITC) is markedly more potent than the natural (DADS) H_2_S donor in the inhibition of inflammatory pain. These effects might be related to the different nature and chemical structure of both compounds [37]. In summary, our data demonstrated the analgesic effects induced by the intraperitoneal administration of P-ITC during inflammatory pain, as was previously demonstrated in other neuropathic and osteoarthritis pain models [6,36]. Furthermore, our data also revealed the analgesic actions of DADS in an inflammatory pain model.

**Figure 9:**
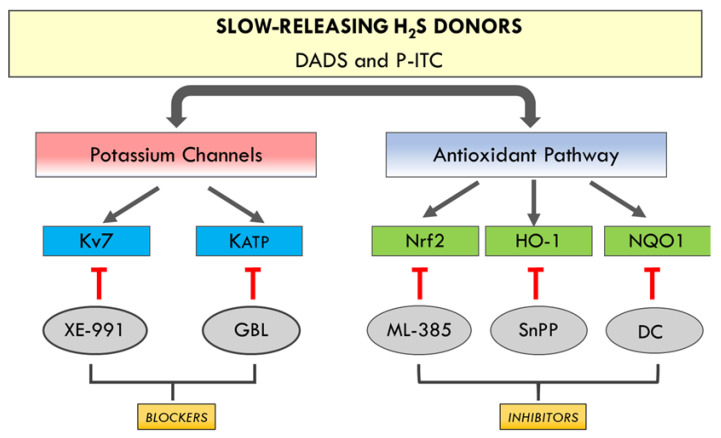
Schematic representation of the proposed mechanism of action of DADS and P-ITC in the inhibition of inflammatory pain. Their analgesic effects are mainly mediated via activating the potassium channels (Kv7 and KATP) and the antioxidant signaling pathway (Nrf2, HO-1 and NQO1).

In addition, the plausible mechanisms involved in the antinociceptive effects induced by DADS and P-ITC in animals with chronic inflammatory pain were evaluated. Particularly, in this study, the involvement of the voltage-gated Kv7 potassium channels in the inhibitory effects of both H_2_S slow-releasers was demonstrated by reversing their antiallodynic and antihyperalgesic effects with the selective Kv7 channel blocker XE-991. Our results further demonstrated the possible participation of KATP potassium channels in the analgesic actions of both H_2_S donors, as their effects were inhibited with the administration of glibenclamide. These data are in agreement with those of other studies, showing the participation of kv7 potassium channels in the antinociceptive effects of other H_2_S donors (A-ITC and GYY4137) during neuropathic and osteoarthritis pain [7,10], and with the implication of the KATP channels in the effects of H_2_S donors in different acute and chronic pain models [11,12]. These findings indicate that the antinociceptive effects of DADS and P-ITC could be mediated via activating Kv7 and KATP potassium channels during inflammatory pain (Figure 9).

In accordance with Guo et al., (2020) [5], our results also demonstrated the reversion of the antiallodynic and antihyperalgesic effects induced by high doses of DADS and P-ITC after administrating selective Nrf2 (ML-385), HO-1 (SnPP), and NQO1 (dicoumarol) inhibitors. These findings suggested that the analgesic actions of DADS and P-ITC during inflammatory pain might also be mediated by activating the Nrf2/HO-1-NQO1 signaling pathway (Figure 9). Accordingly, previous studies reported the implication of the Nrf2/HO-1 pathway in the anti-inflammatory and antinociceptive effects of another H_2_S donor (NaHS) [38], and of DADS in animals with neuropathic pain [10].

Our results confirmed the oxidative stress induced by CFA in the paw, as previously demonstrated [39], with the increased expression of 4-HNE, an oxidative stress marker, in paw tissues. Interestingly, both treatments, DADS and P-ITC, normalized these increased levels of 4-HNE-positive proteins in this tissue, thus revealing the antioxidative properties of both compounds. In accordance with this finding, the administration of other antioxidants, such as oxindoles, also inhibited the high levels of 4-HNE-positive proteins in the paw of CFA-injected mice [39].

The important role that the enzyme HO-1 plays in modulating several nociceptive, oxidative, and inflammatory pathways involved in the development of chronic pain is well acknowledged [40]. In fact, HO-1 induction produced potent analgesic, antioxidant, and/or anti-inflammatory effects during neuropathic pain [41], peripheral inflammation [28], and diabetic neuropathy [42], while its suppression enhanced the osteoarthritis severity [43]. In this study, we also evaluated the effects of DADS and P-ITC in the expression of antioxidant enzymes in the paws of mice with CFA-induced inflammatory pain. While CFA did not modify the NQO1 levels in the paw, it upregulated the expression of HO-1 and SOD-1 and downregulated the expression of GSTM1. This decreased expression of GSTM1 in the paw confirmed the CFA-induced oxidative stress detected with 4-HNE. Moreover, while both slow-releasing H_2_S donors increased the expression of NQO1, maintained the high levels of HO-1, and normalized GSTM1 expression, only P-ITC preserved the elevated SOD-1 levels. This demonstrates the potent antioxidative effects induced by both H_2_S donors in inflammatory pain. In accordance with our data, other studies performed in animals with neuropathic or osteoarthritis pain also demonstrated the activation of the endogenous antioxidant system by slow-releasing H_2_S donors [7,8]. Our findings confirmed the antioxidant properties of these compounds and suggested that it might be, in part, responsible for their analgesic actions during inflammatory pain.

To assess the role played by DADS and P-ITC in the nociceptive and apoptotic responses induced by peripheral inflammation, we evaluated the expression of p-AKT and BAX in the paws of CFA-injected mice treated with these H_2_S donors. It is well known that the phosphorylation of Akt is involved in the pronociceptive responses induced by chronic pain [44]. In accordance with this, significantly increased p-Akt protein levels were demonstrated in the paw tissues of animals with inflammatory pain. Interestingly, DADS and P-ITC both blocked its local activation, revealing the possible participation of Akt in the analgesic effects of these H_2_S donors in CFA-injected mice. In agreement with our results, P-ITC and DADS also lowered the increased p-Akt protein levels induced by osteoarthritis and neuropathic pain in specific brain areas [7,10]. The neuroprotective properties of DADS and P-ITC in animals with inflammatory pain were also demonstrated with the reversion of the enhanced BAX levels induced by CFA. Similar results were obtained in other models of chronic pain or cardiovascular diseases in which H_2_S induction resulted in a reduction in the apoptotic responses [10,44].

Several studies demonstrated that opioids represent a suitable approach for the treatment of chronic pain, but their effectiveness may be limited, and their long-term use may lead to various side effects [24]. In this study, we demonstrated that the subplantar administration of DPDPE and UFP-512, two DOR agonists, inhibited the mechanical allodynia and thermal hyperalgesia induced by CFA in a dose-dependent manner. These results are consistent with those of previous studies that show the dose-dependent inhibitory effects of the subcutaneous or intrathecal administration of DPDPE in the allodynia associated with diabetic neuropathy [45] or the thermal hyperalgesia caused by mild burn [46], and with the antinociceptive effects induced by the intraperitoneal administration of UFP-512 in animals with inflammatory and neuropathic pain [27]. Our data also show that the potency of DPDPE in terms of inhibiting mechanical allodynia and thermal hyperalgesia induced by inflammatory pain was approximately 1.6 times greater than that of UFP-512. Furthermore, the data revealed that H_2_S induction is capable of enhancing the analgesic effects induced by the local administration of low doses of DOR agonists during peripheral inflammation. That is to say, a significant improvement in the antiallodynic and antihyperalgesic effects of both DOR agonists were demonstrated in mice pre-treated with DADS and P-ITC. The most promising combination in terms of reversing mechanical allodynia was the co-administration of P-ITC and UFP-512; this combination demonstrated a 79.0% inhibition as compared with the 53.9% inhibition of P-ITC plus DPDPE, the 54.7% inhibition of DADS plus DPDPE, and the 57.6% inhibition of DADS plus UFP-512. An enhancement of the antihyperalgesic effects of the DOR agonists induced by DADS and P-ITC was also demonstrated, but in this case, all tested combinations exhibited similar effects. That is to say, about 52–64% inhibition of thermal hyperalgesia was produced by the administration of DADS and P-ITC in combination with DPDPE or UFP-512.

Finally, our data also proved that both H_2_S donors increased DOR expression in paw tissues, thus revealing the capacity of H_2_S to enhance the local protein levels of DOR and demonstrating an interaction between this gas and the opioid system. These data are in agreement with the capacity of several HO-1 (cobalt protoporphyrin IX) or Nrf2 (sulforaphane) activators to enhance the effects and expression of DOR in animals with inflammatory pain [28] or neuropathic pain associated with type 2 diabetes [45]. Therefore, we suggest that the augmented expression of DOR triggered by DADS and P-ITC in the paws of animals with inflammatory pain might play a major role in the improvement of the antinociceptive effects of DOR agonists induced by both H_2_S donors under inflammatory pain conditions. Thus, considering the few side effects associated with DOR agonists, their local administration alone and in combination with DADS or P-ITC might be suitable for the treatment of chronic inflammatory pain.

## 5. Conclusions

In conclusion, this study demonstrates that slow-releasing H_2_S donors inhibit inflammatory pain and improve the antinociceptive effects of DOR agonists. The analgesic actions of DADS and P-ITC are mainly produced by activating the potassium channels (Kv7, KATP) and the Nrf2/HO-1-NQO1 signaling pathway. The administration of DADS and P-ITC blocked oxidative stress, activated the antioxidant pathway, and inhibited the plasticity changes provoked by peripheral inflammation. Moreover, both H_2_S donors expanded the local expression of DOR, which might explain the increased antinociceptive effects produced by the co-administration of DPDPE or UFP-512 with DADS or P-ITC in inflammatory pain. Therefore, this study suggests that the systemic administration of DADS and P-ITC or the local administration of DOR agonists in combination with slow-releasing H_2_S donors should be considered as two new strategies for the management of inflammatory pain.

## Figures and Tables

**Table 1:** Comparison of the potencies (ED_50_) of DADS and P-ITC in the inhibition of mechanical allodynia and thermal hyperalgesia induced by peripheral inflammation.

	Mechanical Allodynia	Thermal Hyperalgesia
DADS	87.3 ± 19.8	82.9 ± 9.6
P-ITC	22.3 ± 5.6	21.6 ± 4.5
Ratio (DADS/P-ITC)	3.9	3.8

Data are expressed as ED_50_ values (µmol/kg) ± SEM (*n* = 6 animals per dose). For each test, the ratio of the ED_50_ values between drugs is indicated.

**Table 2:** Comparison of the potencies (ED_50_) of the subplantar administration of DPDPE and UFP-512 on the inhibition of mechanical allodynia and thermal hyperalgesia induced by peripheral inflammation.

	Mechanical Allodynia	Thermal Hyperalgesia
UFP-512	171.9 ± 19.9	127.8 ± 15.6
DPDPE	109.4 ± 12.6	77.9 ± 16.8
Ratio (UFP-512/DPDPE)	1.6	1.6

Data are expressed as ED_50_ values (µg) ± SEM (*n* = 6 animals per dose). For each test, the ratio of the ED_50_ values between drugs is indicated.

## Data Availability

Data is contained within the article.
